# Assessing the Health Impact of Water Quality Interventions in Low-Income Settings: Concerns Associated with Blinded Trials and the Need for Objective Outcomes

**DOI:** 10.1289/ehp.1510532

**Published:** 2015-12-18

**Authors:** Thomas Clasen, Sophie Boisson

**Affiliations:** 1 Department of Environmental Health, Rollins School of Public Health, Emory University, Atlanta, Georgia, USA; 2 Faculty of Infectious and Tropical Diseases, London School of Hygiene & Tropical Medicine, London, United Kingdom

## Abstract

**Background:**

A dramatic disparity between the results of blinded versus open trial designs has raised questions about the effectiveness of water quality interventions and other environmental interventions to prevent diarrhea, a leading killer of young children in low-income countries.

**Objectives:**

We summarize the results of blinded versus open trials of water quality interventions, describe evidence from a recent placebo-controlled trial in India suggesting that control households were put at risk from their participation, and suggest alternatives to blinded trials that could resolve continued uncertainty about the magnitude of the protective effect of water, sanitation, and hygiene (WASH) interventions without presenting ethical questions.

**Discussion:**

Concerns about reporting bias in open trial designs continue to cause uncertainty about the effectiveness of WASH interventions. However, evidence suggests that despite instructions to the contrary, placebos may encourage control group participants in blinded trials to cease practicing traditional water treatment practices in the mistaken belief that they are protected by an active intervention. Although objective outcomes such as pathogen incrimination, seroconversion, biomarkers, and anthropometry can be helpful, these are often costly, nonspecific, and unsuitable for evaluating programmatic interventions.

**Conclusions:**

Unless researchers can be assured that a placebo will not cause those in a control group to change their behavior in a manner that increases their risk, it is incumbent on researchers to use alternatives. Validated objective measures are needed for assessing the health impact of WASH interventions that are reliable, affordable, and suitable both for research and program evaluation.

**Citation:**

Clasen T, Boisson S. 2016. Assessing the health impact of water quality interventions in low-income settings: concerns associated with blinded trials and the need for objective outcomes. Environ Health Perspect 124:886–889; http://dx.doi.org/10.1289/ehp.1510532

## Introduction

The 2010 Global Burden of Disease (GBD) study surprised many by reporting that drinking water quality was not a risk factor for diarrheal disease (Lim et al. 2012). Its conclusion was based on the generally accepted preference for blinded trials over trials following nonblinded or open study designs (Clasen et al. 2015). Researchers assembled by the World Health Organization (WHO) responded promptly. Although they acknowledged the disparity between results based on study design, they opted to pool results from all studies, blinded and open, citing shortcomings from some of the blinded studies (Wolf et al. 2014). This yielded an overall protective effect, restoring safe drinking water as a priority in public health. Recently, the 2013 GBD study used the results from the WHO-sponsored review with no explanation about why it reversed its previous reliance on blinded trials only (GBD 2013 Risk Factors Collaborators et al. 2015).

Double-blinded, randomized, placebo-controlled trials where both the subject and investigator are unaware of whether the subject is a member of the intervention or control group are often cited as the “gold standard” for epidemiological evidence, offering high potential for causal inference. Assuming the generation and concealment of the allocation sequence and other protections, random allocation of study subjects minimizes the risk of selection bias, and blinding reduces the risk of reporting bias (single blinded) and measurement bias (double or triple blinded).

Although double-blinded, randomized controlled trials (RCTs) are common in drug trials where inert agents can often be formulated, packaged, and presented as placebos, blinding at the participant level can be difficult or impossible in the case of basic environmental health interventions, such as improved water supplies, sanitation, and hygiene (WASH) (Allen et al. 2015).

Among WASH interventions, blinding has been attempted only for household water treatment (HWT) interventions designed to assess the health impact of improved drinking water quality. To date, a total of 10 blinded studies have been reported, 4 comparing home-based chlorine with a placebo (Austin 1993; Boisson et al. 2013; Jain et al. 2010; Kirchhoff et al. 1985) and 6 comparing a plumbed-in or table-top household water filter with a sham filter (Boisson et al. 2010; Colford et al. 2002, 2005, 2009; Hellard et al. 2001; Rodrigo et al. 2011). Half of the trials were conducted in low-income settings with water shown to be fecally contaminated (Austin 1993; Boisson et al. 2010, 2013; Jain et al. 2010; Kirchhoff et al. 1985), whereas the balance took place in high-income countries with water shown or presumed to meet international standards. Poor adherence may have also contributed to a lack of effect (Clasen et al. 2015).

Significantly, although trials of HWT interventions that follow open study designs have reported large and fairly consistent protective effects on diarrhea, only one blinded trial has reported the intervention to prevent the disease (Colford et al. 2009). In a recent systematic review and meta-analysis of these studies, the pooled estimate of effect from open-trial designs yielded a relative risk of diarrhea of 0.55 [95% confidence interval (CI): 0.44, 0.68; 46 comparisons], whereas that from blinded trials was 0.94 (95% CI: 0.84, 1.06; 10 analyses) (Clasen et al. 2015). Though questions were raised about compliance and ambient risk, the review concluded that the blinded trials presented little risk of bias.

This disparity in results between open and blinded trials has important questions that are still the subject of much debate in the WASH sector. Some focused specifically on HWT, arguing that that the evidence does not support efforts to scale up the intervention (Schmidt and Cairncross 2009). Others extended the results to water quality interventions generally, concluding that water quality is not a risk factor in the global burden of disease (Engell and Lim 2013; Lim et al. 2012). Still others have generalized the results to all nonblinded studies of household-level WASH interventions. This has led to the conclusion that, after adjusting for the bias due to nonblinding, hygiene interventions are not effective against diarrhea (Freeman et al. 2014).

In our most recent trials of HWT interventions, we used placebos to minimize the risk of reporting bias. A trial in the Democratic Republic of the Congo (DRC) where a sham filter shown to have no effect in the lab actually removed 90% of fecal indicator bacteria in the field (probably due to accumulated biofilm) demonstrated the challenge of implementing a neutral placebo in the case of a filter (Boisson et al. 2010). However, our trial of a chlorine intervention in India raised potentially more fundamental questions about the ethics of blinding trials of WASH interventions in low-income settings (Boisson et al. 2013).

The India trial sought to assess the impact of an intervention consisting of the free distribution of sodium dichloroisocyanurate (NaDCC) tablets and the promotion of their use in bi-monthly household visits (Boisson et al. 2013). NaDCC tablets have long been used for the emergency treatment of water and more recently for the routine treatment of drinking water. This product was selected for the intervention because chlorine is widely used for water treatment and because a previous study reported that blinding of NaDCC tablets was feasible (Jain et al. 2010). The study was conducted in the State of Orissa among 11 informal settlements in the capital city of Bhubaneswar and 20 rural villages in the district of Dhenkanal. Most households rely on water from poorly protected open hand-dug wells, or from yard or public taps connected to a distribution system drawing from wells. At baseline, 30% of households reported treating their water at home, two-thirds of these by boiling.

During enrollment, we sought to have study participants understand the nature of a placebo and the risks of discontinuing potentially effective water management practices. We explained that half of study households would be receiving a tablet that would have no impact on water quality, and that it was unlikely that they would be able to tell the active from the inactive tablet. We encouraged householders to continue their existing water treatment practices throughout the duration of the trial even if they thought they were receiving the active disinfectant. Enumerators reinforced that message during subsequent household visits.

**Figure 1:**
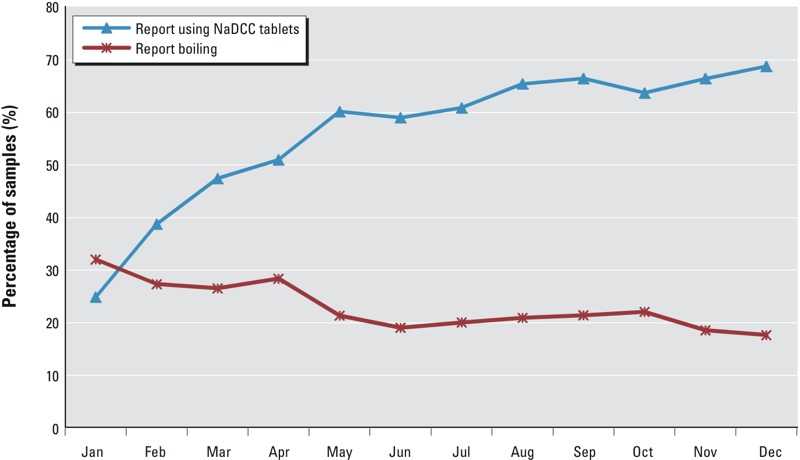
Reported use of tablets and boiling over the 12-month follow-up period (*n* = 22,884 visits).

Despite those recommendations and reminders, Figure 1 shows that some study households gradually switched from boiling to use of the tablets. At the end of the 12-month trial, self-reported boiling decreased by about one-third.

## Discussion

The decision to switch from boiling to use of tablets may have been attributable to participants’ belief that they were part of the intervention arm using active NaDCC tablets. In a post-trial assessment of the effectiveness of blinding, the overwhelming majority of both study arms guessed that they had been assigned to the intervention group (71.5% of intervention group members and 71.2% of control group); only 2.5% of intervention group members and 3.7% of control group members guessed that they were part of the control group (Boisson et al. 2013). Other trials that have reported on the effectiveness of blinding have also found that most study participants (including control group members) believed they had the active intervention (Boisson et al. 2010; Colford et al. 2002, 2009; Jain et al. 2010).

The continuing uncertainty over the effectiveness of water quality interventions satisfies the basic ethics requirement of equipoise in research. However, additional protections are imposed in the case of placebo-controlled trials. According to the Helsinki Declaration (http://www.wma.net/en/30publications/10policies/b3/), unless the placebo represents the “best proven intervention” (i.e., standard of care), a placebo-controlled trial may be undertaken only where “for compelling and scientifically sound methodological reasons the use of placebo is necessary to determine the efficacy or safety of an intervention and the patients who receive placebo or no treatment will not be subject to any risk of serious or irreversible harm.” Although our results from India are not dispositive, they suggest that members of the control group were indeed put at increased risk as a result of discontinuing boiling their water in favor of using the placebo.

The potential for increased risk to the control group raises serious questions about the continued use of a placebo to assess HWT interventions, at least in settings where the water presents known risks and in which members of study population are reported to be practicing a potentially effective alternative such as boiling. Because evidence suggests that most participants in blinded HWT trials believe they have been assigned to the active intervention group, it is not clear that this risk could be mitigated even by carefully cautioning participants to continue their water management practices or reinforcing this message throughout the trial. Such close monitoring may engender reactivity that could undermine study validity (Zwane et al. 2011). It is also unlikely that risks for waterborne disease could be managed through normal surveillance and referral of cases for treatment.

We emphasize that discontinued practice of a potentially protective behavior by the control group does not affect the validity of placebo-controlled trials or their potential to estimate the effect of the intervention. Nevertheless, the ethical issues presented should limit the circumstances in which researchers may undertake placebo-controlled trials of water quality interventions when researchers cannot be sure that members of the control group will not be put at increased risk. At the same time, they should encourage further research to improve the reliability of outcomes in open trial designs.

One approach is to attempt to adjust results for lack of blinding at the participant level. Recent systematic reviews have shown that the nonblinded trials with subjective outcomes may exaggerate effectiveness by around 30% compared to blinded trials (Savović et al. 2012). These results have been used in other reviews of WASH interventions to adjust pooled estimates of effect (Wolf et al. 2014). The adjustment, however, is derived mainly from clinical studies of drug therapies. It is unclear whether the same discount can be applied to field trials of water quality and other WASH interventions or that the adjustment would be homogenous across different populations, interventions, and data collection methods.

Another approach is to exclude households that treat their water (or practice a relevant WASH behavior) as part of the study’s eligibility criteria. This presents at least three problems. First, identifying those that actually practice the behavior is challenging, both because of courtesy and social desirability bias (exaggerating the practice) and the tendency to misreport in order to qualify for study participation. Second, householders exaggerate the consistency of environmental behaviors such as treating their drinking water or collecting it from safe sources (Rosa et al. 2014). Third, excluding self-reported practitioners of these behaviors limits the potential of these studies to estimate larger population effects.

Perhaps the best alternative to blinding at the study participant level is to use objective outcomes. Studies have evaluated the effectiveness of HWT and other WASH interventions by assaying stools for enteric pathogens or blood for seroconversion to waterborne pathogens (Crump et al. 2007; Mahfouz et al. 1995; Quick et al. 1999). More recent studies include potential biomarkers such as inflammatory cytokines or markers of intestinal dysfunction, including environmental enteropathy (Arnold et al. 2013). One-step immunochromatographic dipstick tests have been successfully developed for cholera (Nato et al. 2003) and various Shigella species (Duran et al. 2013). A “lab-on-a-card” diagnostic tool against a broader range of infectious agents responsible for pneumonia and diarrhea—the major killers of young children in low-income countries—would have obvious clinical value. Other studies have used anthropometry—a potential marker of recent (weight-for-age) or longer-term (stunting) enteric infection. Three studies assessed weight-for-age, a potential proxy for acute diarrhea in HWT trials (Boisson et al. 2013; du Preez et al. 2011; Peletz et al. 2012); others have used anthropometry to assess the health impact of sanitation (Arnold et al. 2010; Clasen et al. 2014; Patil et al. 2014). However, caution must be exercised to ensure the objectivity of this metric (Arnold et al. 2012). Other studies have attempted to minimize bias by using clinical records of morbidity and mortality (Mahfouz et al. 1995).

None of the existing objective metrics, however, are wholly satisfactory, especially for evaluating large-scale programs involving WASH interventions. Assays of stools are costly and intrusive, and often are unable to incriminate pathogens. Blood draws for seroconversion studies are also intrusive and often inconclusive even in children in low-income settings where seroprevalence is high even at an early age. The value of anthropometry to assess WASH interventions is still unclear. And reliance on clinical records typically requires much larger sample sizes, and these are subject to other sources of systematic bias.

## Conclusion

Water quality and other WASH interventions have the potential to significantly reduce the burden of disease associated with enteric pathogens. There is an ongoing need, however, to rigorously assess their effectiveness in order to optimize programmatic interventions. Our results suggest that blinding trials at the participant level by using placebos in settings where some participants may already be practicing protective measures may increase the risk of exposure among study participants. There may be cases in which researchers can manage this risk without causing reactivity or otherwise affecting the integrity of the study. Alternative approaches are needed, including the development and validation of objective metrics that are reliable, affordable, and suitable both for research and the monitoring and evaluation of WASH programs.
